# Engraftment and proliferation potential of embryonic lung tissue cells in irradiated mice with emphysema

**DOI:** 10.1038/s41598-019-40237-x

**Published:** 2019-03-06

**Authors:** Kazushige Shiraishi, Shigeyuki Shichino, Tatsuya Tsukui, Shinichi Hashimoto, Satoshi Ueha, Kouji Matsushima

**Affiliations:** 1 Department of Molecular Preventive Medicine, Graduate School of Medicine, The University of Tokyo, Tokyo, 113-0033 Japan; 2 Division of Molecular Regulation of Inflammatory and Immune Diseases, Research Institute of Biomedical Sciences, Tokyo University of Science, Noda, 278-0022 Japan; 3 Department of Integrative Medicine for Longevity, Graduate School of Medical Sciences, Kanazawa University, Kanazawa, 920-8641 Japan

## Abstract

Recently, there has been increasing interest in stem cell transplantation therapy, to treat chronic respiratory diseases, using lung epithelial cells or alveolospheres derived from endogenous lung progenitor cells. However, optimal transplantation strategy of these cells has not been addressed. To gain insight into the optimization of stem cell transplantation therapy, we investigated whether lung cell engraftment potential differ among different developmental stages. After preconditioning with irradiation and elastase to induce lung damage, we infused embryonic day 15.5 (E15.5) CAG-EGFP whole lung cells, and confirmed the engraftment of epithelial cells, endothelial cells, and mesenchymal cells. The number of EGFP-positive epithelial cells increased from day 7 to 28 after infusion. Among epithelial cells derived from E13.5, E15.5, E18.5, P7, P14, and P56 mice, E15.5 cells demonstrated the most efficient engraftment. *In vitro*, E15.5 epithelial cells showed high proliferation potential. Transcriptome analyses of sorted epithelial cells from E13.5, E15.5, E18.5, P14, and P56 mice revealed that cell cycle and cell-cell adhesion genes were highly enriched in E15.5 epithelial cells. Our findings suggest that cell therapy for lung diseases might be most effective when epithelial cells with transcriptional traits similar to those of E15.5 epithelial cells are used.

## Introduction

Stem cell transplantation therapy for chronic respiratory diseases, including chronic obstructive pulmonary disease (COPD), is attracting widespread interest in the field of respiratory medicine^1^. COPD is an incurable, progressive lung disease associated with respiratory dysfunction caused by emphysema or chronic bronchitis^2^. Because the currently available treatments for COPD cannot restore the accelerated loss of lung structure and function^2^, a novel therapeutic approach is warranted. Pathologically, emphysematous lungs show alveolar septal destruction, which leads to enlargement of the distal airspaces^3^. Stem cell therapy, involving alveolar wall repopulation or reconstruction in the vacant emphysematous space, is theoretically an efficient and a novel treatment strategy for COPD.

Lung epithelial cells or alveolospheres, derived from induced pluripotent stem (iPS) cells, and endogenous lung progenitor cells are potentially suitable for transplantation therapy to treat chronic respiratory diseases^1^. Several groups have reported the successful differentiation of human iPS cells towards mature lung epithelial cells and alveolospheres^4–6^; however, optimal transplantation strategy using mouse/human iPS-derived cells or endogenous lung progenitor cells has not been sufficiently addressed.

Regarding strategies for the transplantation of stem cells to the lungs, the engraftment of endogenous progenitor cells or iPS-derived cells to injured lungs *in vivo* has been considered difficult^7^. As such, difficulties underlying the successful transplantation of progenitor cells have delayed progress in this field. This problem was partially solved by Rosen *et al*., who reported that intravenously administered murine canalicular-stage fetal lung progenitors can be successfully engrafted into irradiated mice with naphthalene-induced damage to the airway^8^. However, the types of progenitor cells with optimal transplantation efficiency have not been investigated. For fetal cardiomyocytes, the developmental stage of transplanted cells was previously shown to be an important factor for the efficient transplantation into the heart^9^. Specifically, transplanted embryonic day 14.5 (E14.5) cardiomyocytes showed better integration and persistence than E9.5 and E18.5 cardiomyocytes^9^. During fetal development, the lungs undergo dynamic cellular and molecular changes^10^, but it remains unclear whether these different stages affect transplantation efficiency in the lungs. Furthermore, the types and the transcriptional signatures of progenitor cells with high potential for lung engraftment or cell proliferation still remain elusive. To gain insight into the optimization of stem cell transplantation therapies, we investigated whether cell engraftment or proliferation potential differs among different lung developmental stages using mouse models of elastase-induced emphysema, in which alveoli are expected to be injured. Moreover, we clarified the *in vitro* proliferation potential and transcriptional signatures of the potent epithelial cell population.

## Results

### Radiation pre-treatment enabled engraftment of lung progenitor cells in mouse models of emphysema

**Figure 1:**
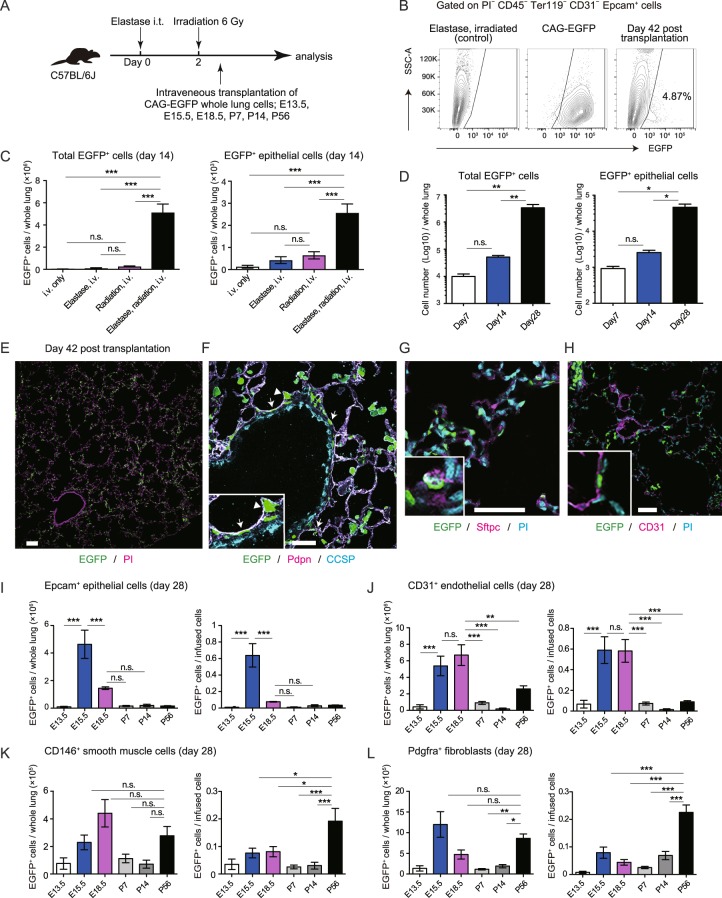
Radiation pre-treatment enables the engraftment of lung progenitor cells in mouse models of emphysema. (**A**) Timeline for mode of two-fold induced damage and progenitor cell treatment; 40−48 h after intratracheal (i.t.) administration of elastase, C57BL/6J recipient mice were irradiated with a sublethal dose. Then, 4–6 h later, animals received whole lung cells from CAG-EGFP E13.5, E15.5, E18.5, P7, P14, or P56 donors. (**B**) Representative flow cytometric plot showing EGFP^+^ Epcam^+^ cells in treated mice (right; day 42 after treatment) compared to that in P56 adult CAG-EGFP mice (middle) and controls (elastase i.t. plus irradiation; left). See Supplementary Fig. S2 for tissue cell identification plots. (**C**) Numbers of EGFP^+^ cells and EGFP^+^ Epcam^+^ epithelial cells on day 14 after treatment. (**D**) Time course analysis of the numbers of EGFP^+^ cells and EGFP^+^ Epcam^+^ epithelial cells on days 7, 14, and 28. (**E**) Lung sections from the treated mice were stained for propidium iodide (PI; magenta). (**F**) Lung sections from the treated mice were stained for Pdpn (magenta) and CCSP (cyan). Arrows indicate Pdpn^+^ EGFP^+^ cells. Arrowhead indicates EGFP^+^ AEC2. (**G**) Lung sections from the treated mice were stained for Sftpc (magenta) and PI (cyan). (**H**) Lung sections from the treated mice were stained for CD31 (magenta) and PI (cyan). (**I**–**L**) Numbers of EGFP^+^ cells and ratios of EGFP^+^ cells/infused cells for epithelial cells, endothelial cells, smooth muscle cells, and fibroblasts. Data represent means ± SEM (n = 5–7 animals) and are representative of two independent experiments. *p < 0.05, **p < 0.01, ***p < 0.001 (compared by one-way ANOVA with Tukey’s post-hoc test). Scale bars: 100 µm (**E**), 50 µm (**F**–**H**).

To determine whether fetal lung progenitors can be engrafted into mouse models of emphysema, and whether these progenitor cells have the potential to reconstruct alveolar walls, we first intratracheally transplanted E15.5 CAG-EGFP total lung cells or sorted Epcam^+^ cells into elastase-treated mice, however, it did not yield efficient engraftment (Supplementary Fig. S1A, S1B). Thus we next intravenously transplanted E15.5 CAG-EGFP total lung cells^8^ into irradiated mice with elastase-induced emphysema in which we adopted elastase instead of naphthalene in the protocol described by Rosen *et al*. (Fig. 1A). This two-fold approach of preconditioning enabled the engraftment of EGFP^+^ cells into the emphysematous lungs (Figs 1B and S2). This method of preconditioning significantly increased the number of engrafted cells compared to that in mice treated with only elastase or irradiation (Fig. 1C). This also enabled us to quantitatively analyze the engraftment potential of infused cells. Time-course analysis revealed a significant increase in the number of engrafted cells after infusion, from day 7 to day 28 (Fig. 1D), suggesting that donor cells proliferated after engraftment. Engrafted EGFP^+^ cells were found to be distributed in the lung parenchyma (Fig. 1E), and these cells included alveolar epithelial cells (Pdpn^+^, Sftpc^+^; Fig. 1F,G) and endothelial cells (CD31^+^; Fig. 1H). We did not observe any EGFP^+^ club cell secretory protein^+^ (CCSP) club cells, which reside in the bronchi and main bronchus^11,12^, in the treated mice (Fig. 1F). Although engrafted cells proliferated after treatment, no measurable alveolar wall reconstruction as assessed histologically by linear intersection^13^, was observed even 8 weeks after the treatment (data not shown). We also performed infusion of sorted epithelial cells alone, but did not detect clear quantitative engraftment of these cells based on flow cytometry (data not shown).

To investigate whether lung progenitors from different stages of fetal development have more potential to repopulate alveolar walls, we infused 1 × 10^6^ E13.5, E15.5, E18.5, P7, P14, or P56 CAG-EGFP lung cells and compared the engraftment potential on day 28 after the transplantation. The number of engrafted epithelial cells was highest in the E15.5 group (Fig. 1I). The engrafted:infused ratio (the number of engrafted epithelial cells divided by the number of infused epithelial cells) was also higher in the E15.5 group (Fig. 1I). These results indicate that the developmental stage at which progenitor cells are isolated affects transplantation efficiency, and that E15.5 epithelial cells might have a high potential for lung engraftment or proliferation after engraftment. Interestingly, the engrafted:infused ratio of CD31^+^ endothelial cells was high in the E15.5 and E18.5 groups, whereas CD146^+^ smooth muscle cells and Pdgfra^+^ fibroblasts, derived from P56 adult mice, had the highest engrafted:infused ratio (Fig. 1J–L).

### E15.5 alveolar epithelial cells actively proliferate and form lung alveolospheres *in vitro*

**Figure 2:**
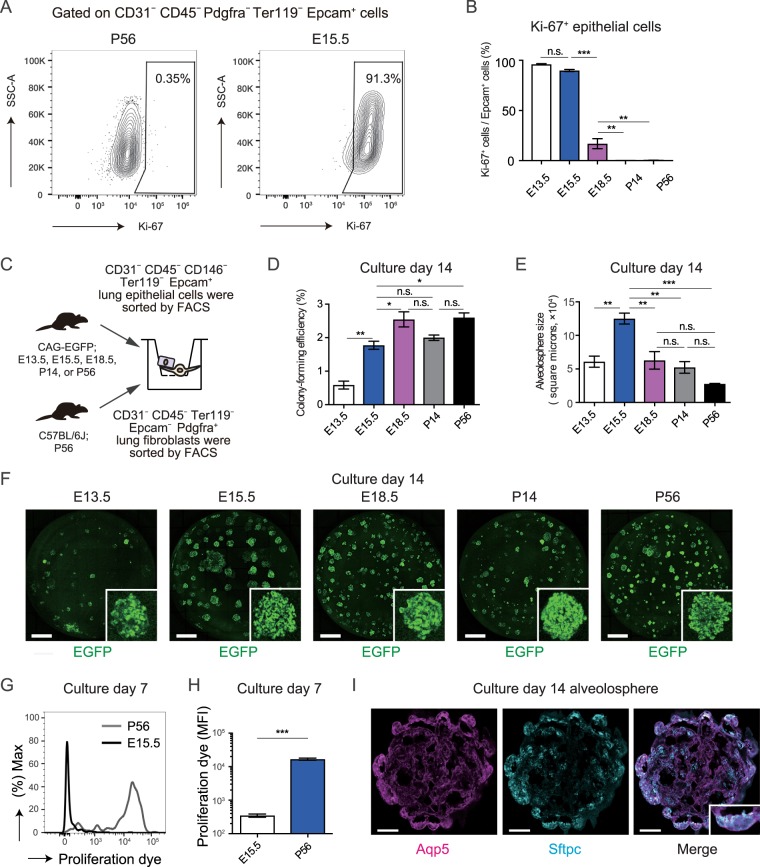
E15.5 epithelial cells actively proliferate and form lung alveolospheres *in vitro*. (**A**) Representative flow cytometry plot showing Ki-67^+^ Epcam^+^ cells in P56 (left) or E15.5 (right) C57BL/6J mice. (**B**) Ki-67-positive cells (percent) per Epcam^+^ epithelial cells in E13.5, E15.5, E18.5, P14, or P56 C57BL/6J mice. Data represent mean ± SEM (n = 3 animals) and are representative of two independent experiments. (**C**) Experimental scheme of the alveolosphere forming assays. E13.5, E15.5, E18.5, P14, or P56 CAG-EGFP epithelial cells were co-cultured with P56 fibroblasts. (**D**,**E**) Colony-forming efficiency (**D**) and size (**E**) of alveolospheres derived from CAG-EGFP epithelial cells during different stages of development. Data represent mean ± SEM (n = 3 wells) and are representative of two independent experiments. (**F**) Representative lung organoids derived from different stages of CAG-EGFP epithelial cells (green) on culture day 14. Z-stack images were reconstructed in 2D. (**G**) Representative histogram showing the intensity of proliferation dye fluorescence detected by flow cytometry. (**H**) Mean fluorescence intensity (MFI) of the proliferation dye. Data represent mean ± SEM (n = 3 wells) and are representative of two independent experiments. (**I**) Representative alveolospheres derived from E15.5 CAG-EGFP epithelial cells (green) were stained for Aqp5 (magenta) and Sftpc (cyan). *p < 0.05, **p < 0.01, ***p < 0.001 [compared by one-way ANOVA with Tukey’s post-hoc test for (**B**,**D**,**E**) and unpaired Student’s t-tests (two-tailed) for (**H**)]. Scale bars: 1 mm (**D**), 50 µm (**E**).

Based on an intravenous transplantation model, it was suggested that the E15.5 epithelial cells repopulate distal alveolus rather than proximal airways after distal lung damage with elastase and radiation, and that alveolar E15.5 epithelial cells might have a high engraftment or proliferation potential among lung epithelial cells derived from different stages of fetal development. To assess the proliferation potential of alveolar E15.5 epithelial cells, we performed Ki-67 staining of E13.5, E15.5, E18.5, P14, or P56 mice lungs. We found that most E13.5 and E15.5 epithelial cells were positive for Ki-67, and the Ki-67-positive epithelial cells decreased as the lung matured (Fig. 2A,B), suggesting that E13.5 and E15.5 might have a higher proliferation potential than E18.5, P14, or P56 epithelial cells. To further evaluate the proliferation potential of alveolar E15.5 epithelial cells, we performed epithelial-alveolar fibroblasts co-culture alveolosphere-forming assays^14^. The rationale for performing the co-culture was to mimic the alveolar stem cell niche *in vitro*, enabling us to estimate epithelial cells proliferation potential after engraftment. Sorted epithelial cells (5 × 10^3^) from E13.5, E15.5, E18.5, P14, or P56 CAG-EGFP mice were co-cultured with Pdgfra^+^ alveolar fibroblasts (1 × 10^5^) sorted from P56 adult C57BL/6J mice (Fig. 2C). Although the colony-forming efficiency was lower in E15.5 epithelial cells than in E18.5 and P56 epithelial cells (Fig. 2D), the size of alveolospheres derived from the E15.5 epithelial cells was largest among all groups (Fig. 2E,F). This suggests that the alveolar epithelial cells derived from E15.5 lungs had the highest potential to expand when cultured with fibroblasts. Indeed, cell proliferation dye-labeling assays revealed that epithelial cells from E15.5 lungs divided more than those from P56 mice 7 days after co-culture with fibroblasts (Fig. 2G,H). Immunohistochemical analysis revealed that alveolospheres derived from E15.5 epithelial cells mostly comprised of Aqp5^+^ and Sftpc^+^ cells (Fig. 2I)^14^, suggesting that the examined spheres were derived from alveolar epithelial cells. Collectively, E15.5 epithelial cells showed a high proliferation potential and formed the largest alveolospheres when co-cultured with fibroblasts, which might reflect their proliferative potential after engraftment.

### Time-course transcriptome analysis of the developing lung identified genes associated with engraftment and proliferation potential

**Figure 3:**
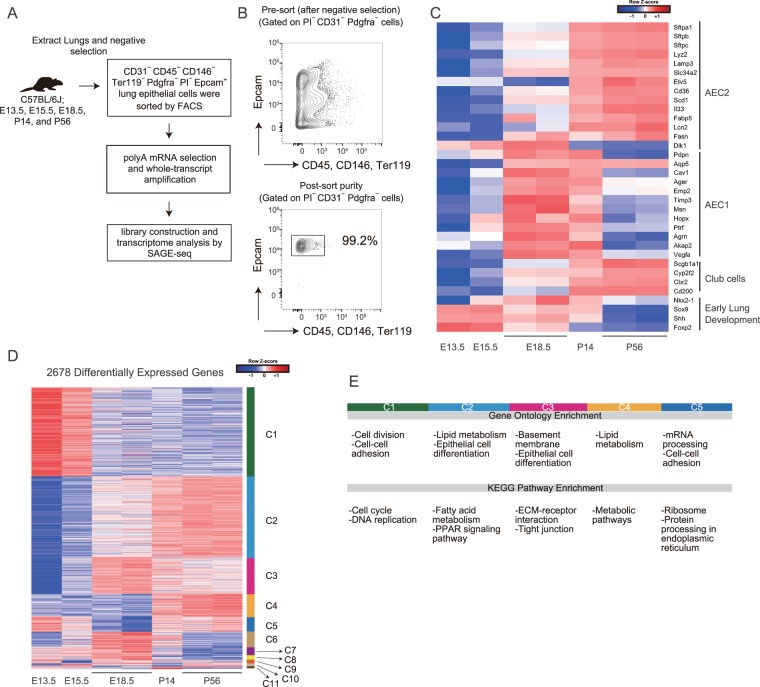
Time-course transcriptome analysis of the developing lung reveals 11 distinct clusters of differentially expressed genes. (**A**) Experimental scheme of transcriptomic analysis of E13.5, E15.5, E18.5, P14, and P56 lung epithelial cells. (**B**) Gating scheme for lung epithelial cells and purity of epithelial cells after cell sorting. Representative plots of P56 mice are shown. (**C**) Heatmap of selected AEC2 markers, AEC1 markers, club cell markers, and early lung development-associated genes. (**D**) Heatmap of the 2678 differentially expressed genes after clustering. Cluster number is shown on the right. (**E**) GO and KEGG pathway enrichment analyses for each cluster (Supplementary Tables S2, S3).

To identify transcriptional signatures associated with the engraftment/proliferation potential of E15.5 epithelial cells, we performed a time-course analysis of the transcriptome of epithelial cells of the developing lungs. We purified lineage (CD31, CD45, Pdgfra, CD146 and Ter119)^−^ Epcam^+^ lung epithelial cells from E13.5, E15.5, E18.5, P14, and P56 C57BL/6J mice and performed serial analysis of gene expression (SAGE) sequencing (Fig. 3A,B). The expression level of alveolar epithelial cell type 2 (AEC2) marker genes^15^, such as *Sftpc*, *Sftpb*, and *Sftpa1*, as well as club cell marker genes increased in a time-dependent manner (Figs 3C and S3A). Genes associated with early lung development, such as *Sox9* and *Nkx2-1*^16^ were up-regulated in fetal epithelial cells but the expression decreased in a time-dependent manner (Figs 3C and S3A). These results suggest that the transcriptome data robustly represented the associated development and the maturation of epithelial cells.

**Figure 4:**
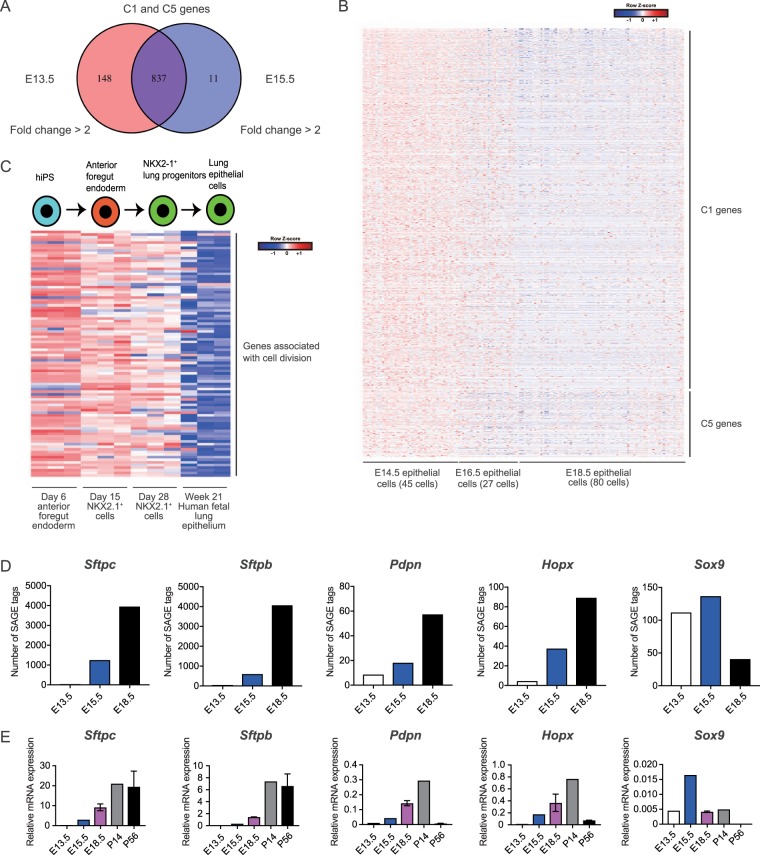
E13.5- and E15.5-specific genes were down-regulated in E18.5 single cells and hiPS-derived mature epithelial cells. (**A**) Venn diagram showing shared C1/C5 genes in E13.5 and E15.5 epithelial cells. (**B**) Heatmap of C1/C5 genes based on the single-cell transcriptome data from E14.5 (45 cells), E16.5 (27 cells), and E18.5 (80 cells) samples^15^. (**C**) Heatmap of the cell division associated genes (GO: 0000278) of C1 after CLICK clustering using expression data from hiPS-derived endoderm, Nkx2-1^+^ lung epithelial progenitor cells, and human fetal lung epithelium^28^. (**D**) The number of SAGE tags (expression level) of the selected AEC2 and AEC1 marker genes and *Sox9*. (E) qPCR of the AEC2 and AEC1 marker genes and *Sox9*; n = 1–2 animals.

We next identified 2678 differentially-expressed genes (DEGs); clustering of the DEGs using the CLICK method^17^ revealed 11 distinct gene clusters (C1−C11; Fig. 3D and Supplementary Table S1). Importantly, genes in cluster C1 and C5 were up-regulated in E13.5 and E15.5 epithelial cells, and gene ontology (GO) and Kyoto Encyclopedia of Genes and Genomes (KEGG) pathway enrichment analysis^18^ revealed that C1 and C5 genes were highly enriched in terms associated with cell division and cell-cell adhesion (Fig. 3E, Supplementary Tables S2, S3). Indeed, focal cell adhesion-related genes were included in C1 and C5 groups, including *Cnn3* and *Zyx*^19^. In addition, well-known cell-cycle associated genes were included in these groups, including *Ran*^20^, *Cdk4*, and *Ccnd1/Cyclin D1*^21^. This suggests that genes associated with cell-cell adhesion genes and cell division might be responsible for the high engraftment and proliferation potential of E15.5 epithelial cells, respectively. Transcription factors were also identified in each cluster (Supplementary Fig. S4 and Supplementary Table S4), and C1 and C5 clusters included well-known cell-cycle regulatory transcription factors such as *E2f1*^22^ and *Tfdp1*^23^. Among the 995 genes identified in the C1 and C5 groups, most were highly expressed in both E13.5 and E15.5 samples (0.5< fold change <2), suggesting that E13.5 and E15.5 epithelial cells share gene expression signatures associated with cell division and adhesion, which decreases with lung development (Fig. 4A). By utilizing the publicly available single-cell transcriptome data of E14.5 (45 cells), E16.5 (27 cells), and E18.5 (80 cells) samples from developing fetal mouse lungs^15^, we confirmed that C1 and C5 genes were up-regulated in E14.5 and E16.5 cells, but were down-regulated in E18.5 cells (Fig. 4B). Interestingly, among the notable alveolar repair associated genes *Tgfb1*, *Tgfb2*, and *Tgfb3*^24^, *Tgfb2* was highly expressed in E13.5 and E15.5 (Supplementary Fig. S5A, B) and was also included in C1. Other notable alveolar repair associated genes *Vegfa*^25^, *Pdgfa*^26^, or *Fgfr2*^27^ were highly expressed in later stages (Supplementary Fig. S5C).

ES/iPS-derived alveolar epithelial cells are expected to be used for lung regeneration. To analyze whether the cell division and cell-cell adhesion associated genes included in C1 were also up-regulated in hiPS-derived alveolar epithelial cells, we utilized available microarray data from cells that were induced to differentiate from hiPS cells into lung epithelial cells^28^. Interestingly, genes associated with cell division, but not cell-cell adhesion (data not shown), were up-regulated in the day-6 anterior foregut endoderm but were gradually down-regulated with maturation to lung epithelial cells (Fig. 4C). These results indicate that cell division-associated genes are shared genes that are expressed early in the pseudoglandular stage (E12.5−E15.5) and canalicular stage (E15.5−E16.5), and are also preserved in early hiPS-derived epithelial cells.

During the fetal lung development, branching morphogenesis (E10−E15^29^) and proximal-distal patterning of the lung slows down around E15.0, and cells in the distal lung begin to express AEC1 and AEC2 markers^16^. The expression levels of AEC2 marker genes *Sftpc/Sftpb* and AEC1 marker genes *Pdpn/Hopx* in E13.5 cells were as low as non-expressed genes, and were lower than those in E15.5 samples (Fig. 4D). *Sox9*, a distal lung epithelial progenitor marker that promotes alveolar epithelial differentiation^30^, was expressed in both E13.5 and E15.5 cells but the expression level was higher in E15.5 samples (Fig. 4D). Finally, we performed quantitative real-time PCR (qPCR) for several AEC1/AEC2 marker genes and *Sox9* to confirm the expression levels observed from SAGE-seq data (Fig. 4E). These findings indicated that E13.5 epithelial cells include Sox9^+^ epithelial progenitor cells but were not matured enough to express AEC2 or AEC1 alveolar cell markers, which may explain why E13.5 cells lack engraftment potential.

## Discussion

To gain insight into the optimization of stem cell transplantation therapy, we showed that E15.5 epithelial cells have maximal engraftment potential *in vivo*, and also exhibit high proliferation potential *in vitro*, compared to those of epithelial cells derived from other developmental stages. We also presented the transcriptional signature of E15.5 epithelial cells, which was found to be highly enriched in cell division- and cell-cell adhesion-associated genes. The transcriptional signatures could explain the high engraftment/proliferation potential *in vivo* as well as the *in vitro* proliferation potential.

We showed that engraftment efficiency differs among lung tissue cell subsets from different developmental stages in elastase/irradiation-damaged lungs. Rosen *et al*. previously showed that infused E15.5 progenitor cells repopulated naphthalene/irradiation-damaged lungs^8^. We first attempted to determine whether repopulated cells could reconstruct damaged alveolar walls using models of emphysema induced by elastase. Contrary to our expectations, we did not find any histologically quantitative changes in alveolar size after treatment. It is possible that infused cells do not reconstruct alveolar structures, but only repopulate the existing lung structure of the recipient mice. Future research should focus on using other animal models of diffuse lung emphysema or using 3D imaging devices to assess treated mice so as to clarify the potential of these cells in the rebuilding of the alveolar wall.

**Figure 5:**
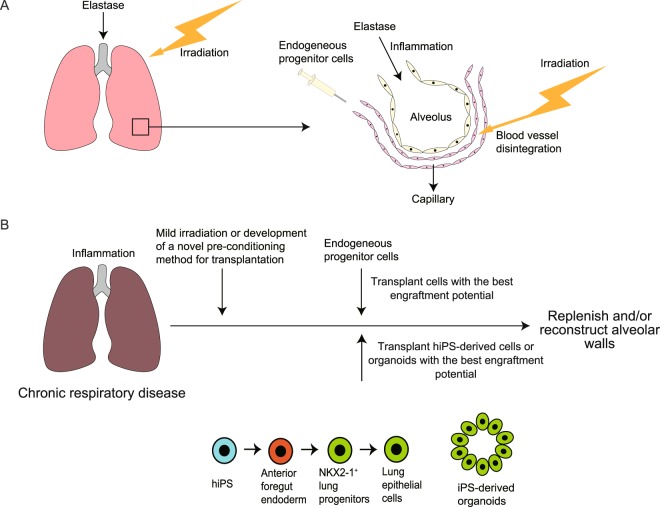
Schematic representation of the transplantation model in this study and potential therapeutic goals in the future. (**A**) Progenitor cells can be engrafted after two-fold elastase-irradiation pre-conditioning. (**B**) Replenishment of epithelial cells with progenitor cells or hiPS-derived cells in patients with chronic respiratory disease might be one of the therapeutic goals in the future.

Rosen *et al*. also reported that infused E15.5 progenitor cells repopulate the lung epithelial cell population, which includes CCSP^+^ bronchiolar club cells^8^. However, we did not observe any CCSP^+^ EGFP^+^ epithelial cells, suggesting that infused cells did not repopulate the proximal epithelial cell population. This discrepancy might be explained by the primary site of lung injury; specifically, naphthalene selectively damages CCSP^+^ club cells that reside in proximal airways^31^, whereas elastase primarily damages the distal lung parenchyma^32^. This interpretation is supported by Nichane *et al*., who found that the lung repopulating potential of *in vitro*-expanded Sox9^+^ lung progenitors differs between bleomycin (parenchyma) and naphthalene (club cells) models^33^. It is admittedly surprising that epithelial cells can be engrafted to the injured lungs after intravenous delivery. We speculate that the irradiation compromises the integrity of blood vessels^34^, allowing the epithelial cells to traverse the compromised blood vessels to seed the lung. Because elastase induces acute inflammation in the lungs^35^ and irradiation compromises the integrity of blood vessels^34^, we presumed that focal lung inflammation in combination with blood vessel disintegration is the key factor for efficient intravenous engraftment of progenitor cells into the lungs (Fig. 5A). However, irradiation can cause comprehensive immunosuppression and systemic damage, which might make the interpretation of transplantation effects complex. The mechanisms underlying cell engraftment should be investigated further in the future studies.

Because the proportion of lung tissue cell subsets from each developmental stage differs, the interpretation of the results in our *in vivo* experiments cannot be completely generalized based on the engraftment potential of single tissue subsets. Clarifying the optimal ratios of epithelial, endothelial, and/or mesenchymal cell mixtures during lung regeneration might also be important to develop novel cell-therapies for COPD.

Moreover, assessing the alveolosphere-formation potential of lung progenitor epithelial cells or ES/iPS-derived epithelial cells might be important to develop and evaluate efficient *in vitro* culture systems for supplying transplantable alveolospheres. We showed that alveolospheres derived from E15.5 epithelial cells were the largest, with evidence of fast cell division. Previously, colon organoids expanded from Lgr5^+^ stem cells were successfully transplanted into the colon epithelium^36,37^, and organoid transplantation into the gastrointestinal lumen is considered a potential future treatment option for patients with inflammatory bowel disease. The protocol for the generation of mouse/human alveolospheres has been established^4–6,14,38^, but the *in vivo* effects of these organoids have not yet been well addressed yet. With regard to regenerative therapy for chronic respiratory diseases, an important question for future studies is to determine if there is any therapeutic effect of lung organoid transplantation. As E15.5 epithelial cell-derived organoids grow faster than those from other epithelial cells, the use of these organoids might accelerate future research in this field.

Our transcriptome analysis revealed gene clusters shared by E13.5 and E15.5 epithelial cells that were highly enriched with cell division and cell-adhesion associated genes. These data could explain the *in vivo* repopulating/proliferation and *in vitro* proliferation potential of E15.5 epithelial cells. With regard to other clusters identified during transcriptome analysis, genes in cluster 2 included the surfactant protein-coding genes *Sftpc*, *Sftpb*, and *Sftpa1*. GO and KEGG pathway enrichment analysis revealed that lipid metabolism, fatty acid metabolism, and PPAR signaling pathways were highly enriched, and we believe that genes in cluster 2 might represent transcriptomic signatures of alveolar epithelial cell maturation and homeostasis. Because club cell markers also showed expression patterns similar to those of cluster 2, club cell homeostatic genes might also be enriched in this cluster. We also identified core transcription factors that were highly expressed in cluster 2, which might regulate alveolar and bronchiolar homeostasis. Cluster 3 included genes that were highly expressed in E18.5 epithelial cells. Interestingly, AEC1 markers^15^ were enriched in cluster 3, and GO and KEGG term analysis also revealed the enrichment of basement membrane, extracellular matrix-receptor interaction, and tight junction terms. This high expression of AEC1 marker genes at E18.5 might represent a transition from alveolar AEC1/AEC2 bipotential cells to AEC1 cells^15^, which could be required to construct alveolar walls. Core transcription factors that might be associated with this process were also identified in this study. These findings could provide insights into future epithelial cell development research.

The reason E13.5 epithelial cells did not show efficient proliferation *in vivo* and *in vitro* is presumed to be their immatureness, which could partially be explained by their low expression of AEC markers. During fetal lung development, branching morphogenesis and proximal-distal patterning of the lung slows around E15.0, and the cells in the distal lung begin to express AEC1 and AEC2 markers^16^. These changes in the expression of AEC1/AEC2 marker genes were confirmed in this study. It has also been proposed that the epithelial branching program antagonizes alveolar differentiation^30^, and this is in accordance with our *in vitro* findings that E13.5 epithelial cells generated few alveolospheres.

Understanding the types and transcriptional signatures of progenitor cells with optimal alveolar repopulating potential could lead to further studies in the field of cell therapy. Although it was not achieved in this study, cell therapy that results in alveolar wall reconstruction could theoretically be used in the future to treat COPD, a disease that involves alveolar wall destruction. Because cell division-associated genes, which are highly expressed genes of E15.5 epithelial cells, were found to be downregulated in mature Nkx2-1^+^ iPS-derived cells, it is possible that successful lung repair strategies involving ES/iPS-derived cells will require the use of early iPS-derived epithelial cells that highly express cell division associated genes. We also speculate that cell therapy for lung diseases in general might be most effective when using epithelial cells with transcriptional traits similar to those of E15.5 epithelial cells (Fig. 5B). Because Tgfb2 was highly expressed in E15.5 epithelial cells, E15.5 epithelial cells might have a better alveolar repairing potential than epithelial cells obtained from other developmental stages.

One of the limitations of our study is that we did not assess functional changes of the lungs after transplantation. Assessment of the lung functions with pulmonary function tests and barrier function evaluation should be considered in the future studies. In addition, functional differences between alveolospheres derived from epithelial cells of different developmental stages were not assessed. It will be very important to assess the functions of spheres before transplantation if alveolospheres are to be considered to be as a new modality for cell transplantation.

In conclusion, E15.5 epithelial cells were shown to have the best engraftment potential *in vivo* and proliferation potential *in vitro*. The transcriptional traits that could explain this potential were lost as these cells differentiate towards mature epithelial cells. Thus, successful lung repair strategies, involving lung progenitor cells or ES/iPS-derived cells will require careful control of cell differentiation stage.

## Methods

### Mice

Female C57BL/6J mice (8–12 weeks of age), and female C57BL/6-Tg(CAG-EGFP)C14-Y01-FM131Osb (CAG-EGFP) mice (8 weeks of age) were purchased from Japan SLC (Shizuoka, Japan). E13.5, E15.5, E18.5, postnatal day 7 (P7), P14, and P56 CAG-EGFP mice were generated in-house by breeding C57BL/6J mice with CAG-EGFP mice. Mice were bred and maintained under specific pathogen-free conditions in the animal facilities at the University of Tokyo. All experiments were approved by the University of Tokyo and Tokyo University of Science, and performed in accordance with the guidelines of the Animal Care Committee of the Graduate School of Medicine, the University of Tokyo and Tokyo University of Science.

### Murine lung cell preparation

For the preparation of donor murine lung cells, E13.5, E15.5, E18.5 fetal lungs and P7, P14, P56 mouse lungs were collected and minced with a sterile razor. Minced lungs were dissociated with Dulbecco’s modified Eagle’s medium (DMEM; Sigma-Aldrich) with 0.96 mg/mL dispase II (Roche), 0.2% collagenase (Wako Pure Chemical Industries), and 20 kU/mL DNase I (Sigma-Aldrich) solution as previously described^39^. After a 60-min incubation at 37 °C, cells were filtered through a 70-µm cell strainer (BD Biosciences), and washed with DMEM supplemented with 2% fetal bovine serum. Erythrocytes were removed by Percoll gradient (70%) centrifugation (GE Healthcare) for 20 min at room temperature (1,000 × g). Single-cell suspensions were further incubated with Ter119-biotinylated (1:200; clone TER-119; BD Biosciences) antibody for 20 min (4 °C), followed by anti-biotin MACS beads (Miltenyi) for 20 min (4 °C). Incubated cells were depleted of erythrocytes through negative selection using an AutoMACS cell separator (Miltenyi Biotech). Resulting propidium iodide staining negative cells were counted using Flow-Count fluorospheres (Beckman Coulter) and a Gallios flow cytometer (Beckman Coulter).

### Antibodies

Fluorochrome- or biotin-conjugated antibodies and streptavidin were purchased from BD Biosciences and BioLegend. Antibodies used for murine lung cell preparation, flow cytometry, and cell sorting are listed in Supplementary Table S5.

### Murine lung cell transplantation model

For transplantation of murine lung cells, recipient C57BL/6J mice were administered with a single dose of porcine pancreas elastase (4U in 50 µL of sterile saline; Elastin Products Co., Inc) by oropharyngeal aspiration, with animals under deep anesthesia as previously described^39^. For intravenous transplantation, elastase-treated mice were further irradiated with 6 Gy after 40−48 h of elastase administration^8^. Irradiated mice were intravenously administered with 1 × 10^6^ donor lung cells via the tail vein within 4−6 h after irradiation. For intratracheal transplantation, elastase-treated mice were intratracheally administered with 1 × 10^6^ donor lung cells or 5 × 10^4^ sorted donor epithelial cells within 40−48 h after elastase treatment.

### Flow cytometry

Flow cytometry analysis was performed to analyze donor cells before and after infusion, recipient lungs after treatment, co-cultured alveolospheres, and Ki-67 positivity of the lungs from different developmental stages. For the flow cytometric analysis of transplanted recipient lungs and Ki-67 positivity, mice were sacrificed and lung single-cell suspensions were collected as described. Flow-Count fluorospheres (Beckman Coulter) and propidium iodide were used to estimate the number of live cells. Single-cell suspensions were blocked with anti-CD16/32 monoclonal antibody for 15 min (4 °C), and stained for 30 min (4 °C) with the antibodies listed in Supplementary Table S5. Stained cells were washed twice with isoflow and passed through a 70-µm cell strainer. For Ki-67 immunostaining, Foxp3/Transcription Factor Fixation/Permeabilization Concentrate and Diluent (Thermo Fisher Scientific) were further used according to the manufacturer’s protocols. Data were acquired for 200,000 to 2 million events using a Gallios flow cytometer (Beckman Coulter) and analyzed with FlowJo software (version 10.4.1; FlowJo LLC).

### Immunohistochemistry

Immunohistochemistry on lungs was performed as described previously^39^. Mouse lungs and alveolospheres were fixed with 4% PFA in PBS and cryosections (10-µm) were prepared. Antibodies used are listed in Supplementary Table S6. All images were acquired using an SP-5 confocal microscope (Leica Microsystems).

### Cell sorting

Cell sorting was performed to purify lung epithelial cells and fibroblasts for lung alveolosphere formation assays and to purify lung epithelial cells for transcriptome analysis. For epithelial cell sorting, single-cell suspensions of lung cells were first stained with anti-mouse CD31, CD45, Ter119, CD146, and Epcam antibodies (see Supplementary Table S5 for details). Cells were next stained with streptavidin-APC, followed by incubation with anti-APC microbeads (Miltenyi). Labeled cells were magnetically depleted. Finally, lineage (CD31, CD45, Ter119, and CD146)^−^ propidium iodide^−^ Epcam^+^ live epithelial cells were sorted using a MoFlo Astrios flow cytometer (Beckman Coulter). For fibroblast cell sorting, single-cell suspensions of lung cells were first stained with anti-mouse CD31, CD45, Ter119, Epcam, and Pdgfra antibodies (see Supplementary Table S5 for details). Cells were stained with streptavidin-APC, followed by anti-APC microbeads (Miltenyi). Lineage (CD31, CD45, Ter119)^−^ propidium iodide^−^ Pdgfra^+^ live fibroblasts were sorted after magnetic depletion.

### Lung alveolosphere formation assay

Epithelial cells (5 × 10^3^) derived from E13.5, E15.5, E18.5, P14, or P56 murine lungs and fibroblasts (1 × 10^5^) derived from P56 murine lungs were sorted into 500 µL of MTEC/Plus medium^40^, resuspended in MTEC/Plus medium, and mixed 1:1 with growth factor–reduced Matrigel (BD Biosciences). The resulting 90 µl mixture of was placed in a 24-well 0.4-μm Transwell clear insert (Falcon; BD Biosciences)^14^. Next, 500 µl MTEC/Plus medium was added to the lower chamber, and MTEC/Plus medium was changed every other day^14^. The Y-27632 ROCK inhibitor (10 μM; Wako Pure Chemical Industries) was added to the medium for the first 2 days. Images were acquired after 14 days of culture. Colony sizes and numbers were quantified using ImageJ version 1.8 (NIH, Bethesda, MD; http://imagej.nih.gov/ij). Colony forming efficiency was defined as the number of colonies formed divided by 5,000 (number of cells plated). For proliferation dye assay, sorted epithelial cells were labeled with Cell Proliferation Dye eFluor 670 (Thermo Fisher Scientific), and 5 × 10^3^ cells were used for co-culture with 1 × 10^5^ fibroblasts derived from P56 murine lungs.

### Construction of SAGE library

Construction of SAGE library was performed as previously described^41,42^. For details of the methods used for library construction of E13.5, E15.5, E18.5, P14, and P56 epithelial cells, please refer to the Supplementary materials and methods. In brief, E13.5, E15.5, E18.5, P14, and P56 epithelial cells (10,000 cells) were directly sorted into 500 µL of cell lysis buffer, and the whole transcripts of the epithelial cells were amplified according to a previous report with modifications^43^. Then, the SAGE library was constructed according to the previous report with modifications^44^. The whole-transcript library was digested with NlaIII (New England Biolabs), and biotinylated transcripts were immobilized onto Dynabeads M-280 streptavidin (Thermo Fisher Scientific). CS1-EcoP15I-NlaIII adapters (for details of primers used for library construction, please see Supplementary Table S7) were then ligated to the transcripts. The resultant transcripts were digested with EcoP15I (New England Biolabs). End repair/A-tailing/ligation reactions were performed using NEBNext Ultra II modules (New England Biolabs) and the CS2-adapter according to the manufacturer’s instructions. For barcoding, the Ion-trP1-CS2 primer and IonA-BC[N]-CS1-primer were added, and PCR was performed. Library concentration was quantified using the KAPA library Quantification Kit for Ion Torrent (KAPA Biosystems).

### Sequencing of SAGE library

RNA sequencing was performed with the Ion Hi-Q Chef Kit, Ion PI v3 Chip Kit and Ion Proton Sequencer (Thermo Fisher Scientific) according to the manufacturer’s instructions. Fastq files were evaluated for Q20 ratios using FastQC and trimmed using Trimommatic-0.33^45^ and PRINSEQ-0.20.4^46^. Trimmed reads were mapped to the Refseq mm10 using Bowtie2-2.2.5^47^ with the following parameters: -t -p 11 -N 1 -D 200 -R 20 -L 20 -i S,1,0.50–norc. Tag numbers of each gene represented the expression level of each gene, and therefore were used as count data. Sample to sample normalization was performed with R (version 3.4.0) and the TCC package^48^. Normalized data were then tested for differential gene expression analysis using the TCC package (integrates edgeR package^49^, with the glmLRT formula and Benjamini-Hochberg correction). Genes with adjusted *P*-values < 0.05 and maximum expression >50 were identified as statistically significant differentially expressed genes. Differentially expressed genes were clustered using the CLICK method^17^. GO and KEGG pathway enrichment analyses were performed using DAVID^18^. Standardized single-cell transcriptome data (GSE52583^15^) for E14.5, E16.5, and E18.5 lung epithelial cells, and standardized microarray data (GSE83310^28^) from experiments on the differentiation of human iPS to Nkx2.1^+^ cells, were downloaded from the NCBI Gene Expression Omnibus (GEO; http:// www.ncbi.nlm.nih.gov/geo).

### qPCR analysis

qPCR was performed using Thunderbird SYBR qPCR Mix (Toyobo, Osaka, Japan) for the detection of amplified products, with an ABI 7500 real-time PCR system (Life Technologies). A portion of the cDNA obtained from SAGE analysis was used as the template. The expression levels of the mRNAs were normalized to the expression level of *Gapdh* in each sample. Primers used for qPCR are shown in Supplementary Table S8.

### Statistical analyses

For figures depicting flow cytometry and colony formation assays, data are shown as the mean ± standard error. Differences between means of measurements were tested by unpaired Student’s *t*-tests (two-tailed). For differences in the means among >2 groups, one-way analysis of variance with the Tukey-Kramer’s multiple comparisons *post-hoc* test was performed. *P* < 0.05 was considered statistically significant. Statistical analysis was performed using the GraphPad Prism software version 5.01 (GraphPad Software).

### Accession codes

Raw data from the SAGE-seq have been deposited in the GEO (Accession GSE109847). Part of the data was used in our previous study^42^.

## Supplementary information

Supplementary Information and Figures

Supplementary Tables S1, S2, S3, S4, S5, S6, S7, and S8

## Data Availability

All data generated or analyzed during this study are included in this published article or its Supplemental Information files.
