# Supplementary material for: Engraftment and proliferation potential of embryonic lung tissue cells in irradiated mice with emphysema
Source: Sci Rep. 2019 Mar 6;9:3657. doi: 10.1038/s41598-019-40237-x (PMC6403395; doi:10.1038/s41598-019-40237-x)
Supplement: Supplementary file 1 — Supplementary Information and Figures [file 41598_2019_40237_MOESM1_ESM.pdf]

Engraftment and proliferation potential of embryonic lung tissue cells in irradiated mice with emphysema

Kazushige Shiraishi[1,2], Shigeyuki Shichino[1,2], Tatsuya Tsukui[1], Shinichi Hashimoto[1,2,3], Satoshi Ueha[1,2], Kouji Matsushima[1,2,*]

[1]Department of Molecular Preventive Medicine, Graduate School of Medicine, The University of Tokyo, Tokyo 113-0033, Japan

[2]Division of Molecular Regulation of Inflammatory and Immune Diseases, Research Institute of Biomedical Sciences, Tokyo University of Science, Noda 278-0022, Japan

[3]Department of Integrative Medicine for Longevity, Graduate School of Medical Sciences, Kanazawa University, Kanazawa 920-8641, Japan

*Correspondence: koujim@rs.tus.ac.jp

Supplementary Information (Materials and Methods)

Construction of serial analysis of gene expression (SAGE) library

E13.5, E15.5, E18.5, P14, and P56 epithelial cells (10,000 cells) were sorted in 500 µL cell lysis buffer composed of 100 mM Tris-HCl (pH 7.5), 1% lithium dodecyl sulfate (Sigma-Aldrich), 500 mM lithium chloride, 10 mM ethylenediaminetetraacetic acid (EDTA), and 5 mM dithiothreitol (DTT; Thermo Fisher Scientific). Whole transcripts of epithelial cells were amplified according to a previous report with modifications[1]. In brief, 0.5 pmol biotin-TEG-adapter-dT25 primers (sequences of the primers are shown in Supplementary Table S7) were bound onto 20 µL of Dynabeads M270 streptavidin (Thermo Fisher Scientific). Washed beads were added to each cell lysate, and incubated for 30 min at room temperature. The beads were resuspended in a solution of 2 mM dNTP in 10 µL of reverse transcription mix 1 composed of 1× SuperScript IV buffer (Thermo Fisher Scientific), 2 M betaine (Sigma-Aldrich), 12 mM $MgCl_2$, and 3.2 U/µL RNaseIn Plus (Promega) followed by incubation for 90 s at 70°C and 5 min at 35°C. For the reverse transcription reaction, 10 µl of reverse transcription mix 2 composed of 1× SuperScript IV buffer, 10 mM DTT, 10 U/µL SuperScript IV (Thermo Fisher Scientific), and 2 M betaine was added followed by incubation for 5 min at 35°C and for 15 min at 50°C. To digest the reverse-transcribed mRNA, 20 µL of RNase H mix composed of 1× first-strand buffer (Life Technologies), 5 mM DTT, and 0.6 U RNase H (Thermo Fisher Scientific) was added followed by incubation for 20 min at 37°C. For polyA-tailing, 20 µL terminal deoxynucleotidyl transferase (TdT) mix composed of 50 mM Tris-HCl (pH 8.0), 100 mM KCl, 1 mM $CoCl_2$ (Roche), 3 mM $MgCl_2$, 0.65 mM dATP (Thermo Fisher Scientific), and 15.2 U/µL TdT (Roche) was added followed by incubation for 2 min at 37°C. The reaction was terminated by adding 5 µL of 0.5 M EDTA. The beads were washed, and 20 µL of 2nd strand synthesis mix composed of 1× KAPA Hifi ReadyMix (KAPA Biosystems) and 0.4 µM anchored tagging primer was added; 2nd strand synthesis was carried out with the following program: 95°C for 2 min, 98°C for 20 s, 44°C for 2 min, and 72°C for 7 min. The beads were washed, and the first round of whole-transcript amplification (WTA) was performed with the 25 µL first WTA mix composed of 1× KAPA Hifi ReadyMix, 0.4 µM 3' WTA primer, and 0.4 µM anchored tagging primer with the following program: 95°C for 3 min; seven cycles at 98°C for 20 s, 65°C for 15 s, and 72°C for 7 min; and 72°C for 5 min. The PCR product was purified with AmPure XP beads (Beckman Coulter). The second WTA mix composed of 1× KAPA Hifi ReadyMix, 0.614 µM biotin-TEG-3' WTA primer, and 0.614 µM 5' WTA primer was added, and the second round of WTA was performed with the following program: 95°C for 3 min; nine cycles at 98°C for 20 s, 65°C for 15 s, and 72°C for 7 min; and 72°C for 5 min. The PCR product was purified using AmPure XP beads.

The SAGE library was constructed according to a previous report with modifications[2]. In brief, 100 ng of the whole-transcript library was digested with NlaIII (New England Biolabs) for 2 h at 37°C, and biotinylated 3′-tail transcripts were immobilized onto Dynabeads M-280 streptavidin (Thermo Fisher Scientific). Next, 10 pmol of CS1-EcoP15I-NlaIII adapters was ligated using the Mighty Mix DNA ligation kit (Takara Bio) for 30 min at 16°C. The beads were resuspended in 200 µL of EcoP15I digestion mix composed of 1× NEBuffer 3.1, 0.2 U EcoP15I (New England Biolabs), and 1 mM ATP and digested for 16 h at 37°C. End repair/A-tailing/ligation reactions were performed using NEBNext Ultra II modules (New England Biolabs) and 1.875 pmol of CS2-adapter according to the manufacturer's instructions. The Qiagen MinElute Column (Qiagen) was used to purify the reaction and elution was

performed with 13 μL of water. For barcoding, 14.25 μL of a mixture composed of 0.614 μM IonA-BC[N]-CS1-primer, 0.614 μM Ion-trP1-CS2 primer, and 1× KAPA Hifi ReadyMix was combined with 10.75 μL of eluate. Then, PCR was performed using the following program: 98°C for 45 s; nine cycles at 98°C for 15 s, 65°C for 30 s, and 72°C for 90 s; and 72°C for 1 min. Library concentration was quantified using the KAPA Library Quantification Kit for Ion Torrent (KAPA Biosystems). SAGE libraries were pooled and the concentration was adjusted to 100 pM.

References

1. Huang, H. *et al.* Non-biased and efficient global amplification of a single-cell cDNA library. *Nucleic Acids Res.* **42,** e12–e12 (2014).
2. Matsumura, H. *et al.* SuperSAGE: powerful serial analysis of gene expression. *Methods Mol. Biol.* **883,** 1–17 (2012).

Supplementary Figure S1

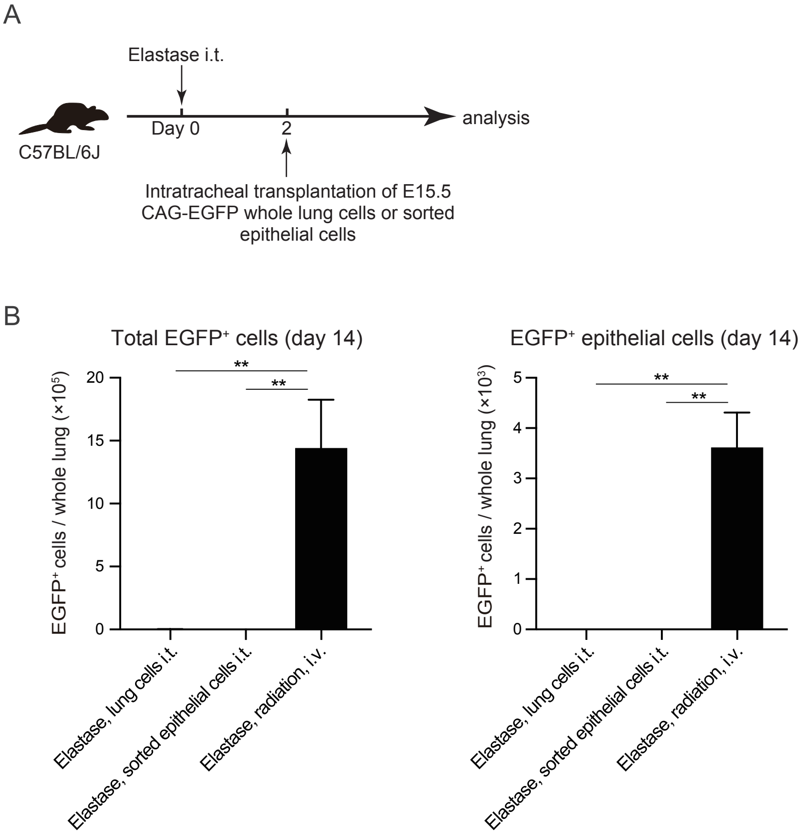

Supplementary Figure S1. Intratracheal cell transfer did not engraft progenitor cells into mouse models of emphysema. (A) Timeline for mode of elastase induced damage model and intratracheal (i.t.) progenitor cell treatment; 40–48 h after i.t. administration of elastase, C57BL/6J recipient mice received i.t. treatment of 1×10^6 whole lung cells or 5×10^4 sorted epithelial cells from CAG-EGFP E15.5 donors. (B) Numbers of $EGFP^+$ cells and $EGFP^+$ $Epcam^+$ epithelial cells on day 14 after treatment. Intravenous administration of whole lung cells after two-fold induced damage was considered positive control. Data represent means ± SEM (n = 3 animals) and are representative of two independent experiments. $^{**}p < 0.01$ (compared by one-way ANOVA with Tukey's post-hoc test).

Supplementary Figure S2

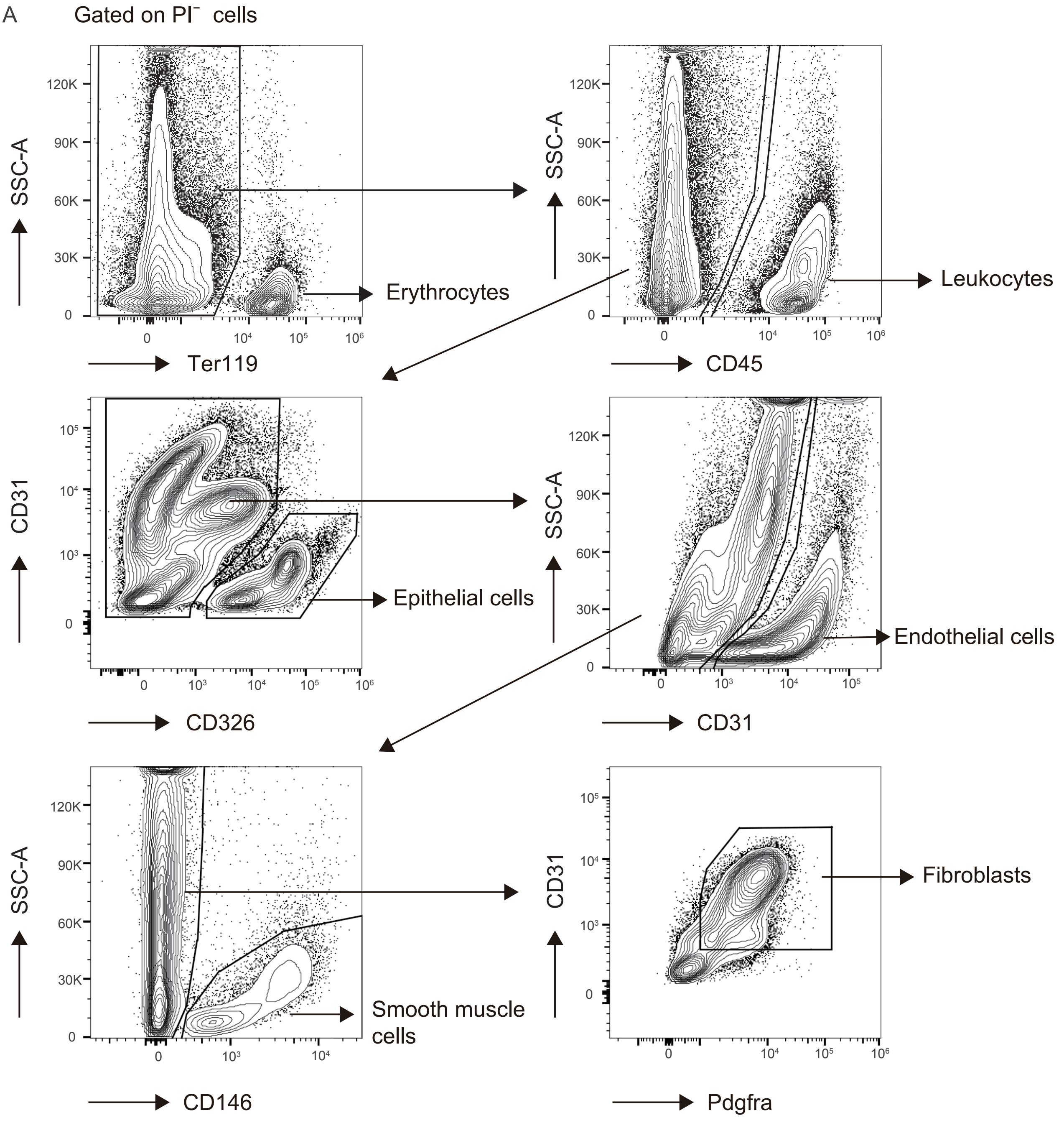

Supplementary Figure S2. Identification of tissue cells by flow cytometry. (A) Gating scheme for whole lung cell subsets. Single-cell suspensions from the whole lung were stained with antibodies. Representative flow cytometric plots showing the gating scheme for lung leukocytes, epithelial cells, endothelial cells, smooth muscle cells, and fibroblasts.

Supplementary Figure S3

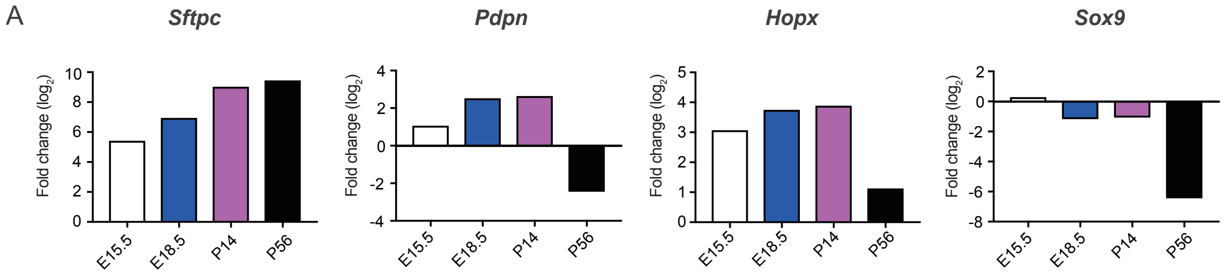

Supplementary Figure S3. Fold change expression of selected genes. (A) Fold change expression of *Sftpc*, *Pdpn*, *Hopx*, and *Sox9* in the transcriptome data. Data are presented as the mean Log_2-fold change in expression compared to E13.5.

Supplementary Figure S4

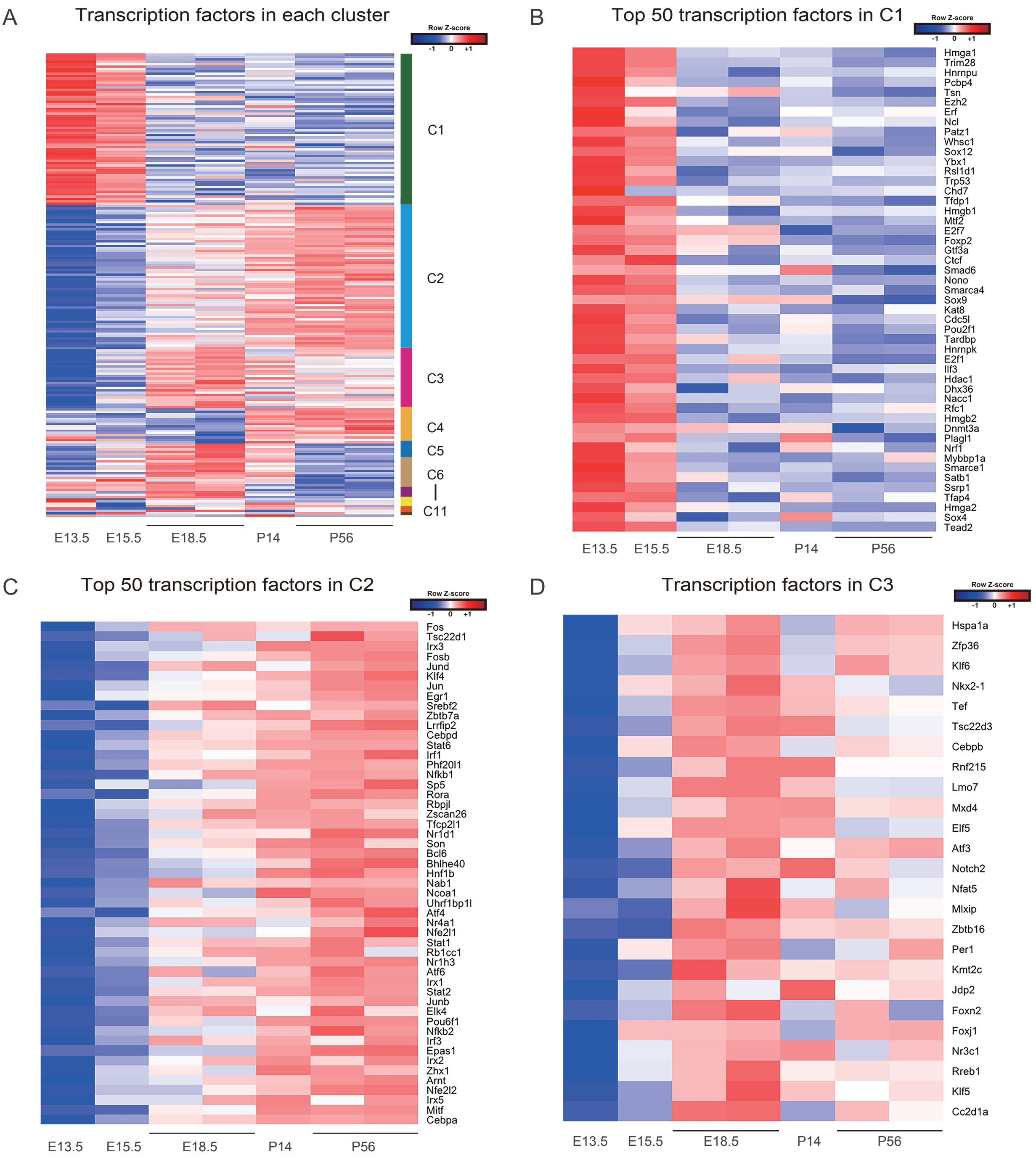

Supplementary Figure S4. Transcriptomic profiling of transcription factors in the developing lungs. (A) Heatmap of the transcription factors differentially expressed in the developing lungs after clustering. Cluster number is shown on the right. (B) Heatmap of the top 50 transcription factors in cluster 1 (ranked by absolute SAGE tag number). (C) Heatmap of the top 50 transcription factors in cluster 2 (ranked by absolute SAGE tag number). (D) Heatmap of the transcription factors in cluster 3.

Supplementary Figure S5

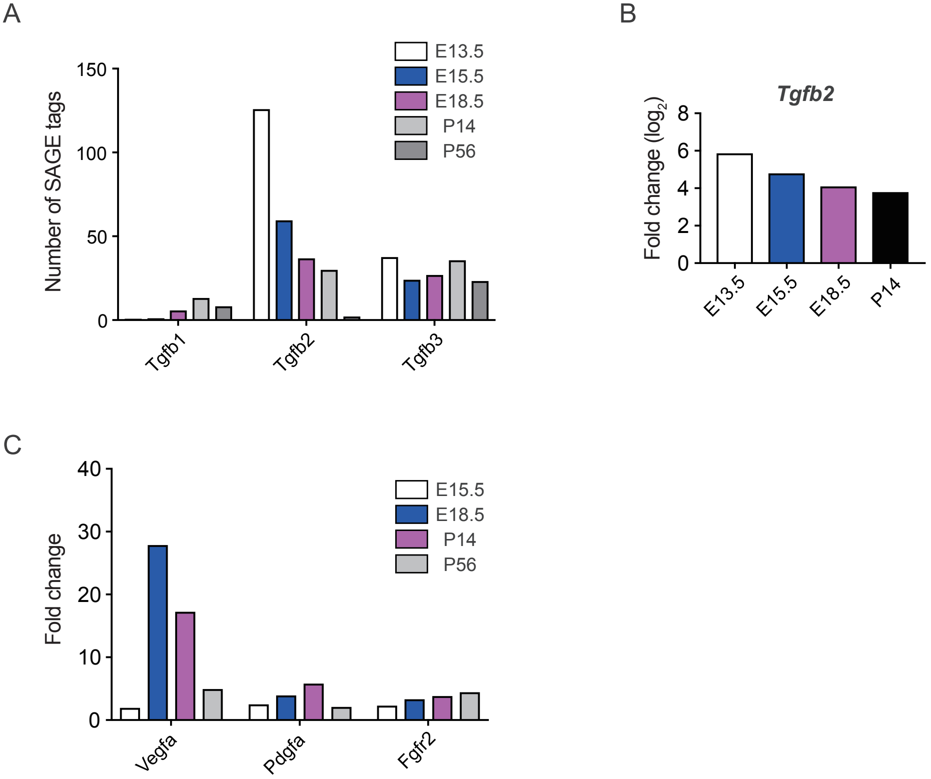

Supplementary Figure S5. Fold change expression of alveolar repair-associated genes. (A) The number of SAGE tags (expression level) of *Tgfb1*, *Tgfb2*, and *Tgfb3* in the transcriptome data. Data are presented as mean expression. (B) Fold change expression of *Tgfb2* in the transcriptome data. Data are presented as Log_2-fold change in expression compared to P56. (C) Fold change expression of *Vegfa*, *Pdgfa*, and *Fgfr2* in the transcriptome data. Data are presented as fold change in expression compared to E13.5.
